# Expression of early transcription factors Oct-4, Sox-2 and Nanog by porcine umbilical cord (PUC) matrix cells

**DOI:** 10.1186/1477-7827-4-8

**Published:** 2006-02-06

**Authors:** Ryan Carlin, Duane Davis, Mark Weiss, Bruce Schultz, Deryl Troyer

**Affiliations:** 1 Dept of Anatomy & Physiology, Kansas State University, Manhattan, KS, USA; 2 Dept of Animal Science and Industry, Kansas State University, Manhattan, KS, USA

## Abstract

**Background:**

Three transcription factors that are expressed at high levels in embryonic stem cells (ESCs) are Nanog, Oct-4 and Sox-2. These transcription factors regulate the expression of other genes during development and are found at high levels in the pluripotent cells of the inner cell mass. The downregulation of these three transcription factors correlates with the loss of pluripotency and self-renewal, and the beginning of subsequent differentiation steps. The roles of Nanog, Oct-4 and Sox-2 have not been fully elucidated. They are important in embryonic development and maintenance of pluripotency in ESCs. We studied the expression of these transcription factors in porcine umbilical cord (PUC) matrix cells.

**Methods:**

Cells were isolated from Wharton's jelly of porcine umbilical cords (PUC) and histochemically assayed for the presence of alkaline phosphatase and the presence of Nanog, Oct-4 and Sox-2 mRNA and protein. PCR amplicons were sequenced and compared with known sequences. The synthesis of Oct-4 and Nanog protein was analyzed using immunocytochemistry. FACS analysis was utilized to evaluate Hoechst 33342 dye-stained cells.

**Results:**

PUC isolates were maintained in culture and formed colonies that express alkaline phosphatase. FACS analysis revealed a side population of Hoechst dye-excluding cells, the Hoechst exclusion was verapamil sensitive. Quantitative and non-quantitative RT-PCR reactions revealed expression of Nanog, Oct-4 and Sox-2 in day 15 embryonic discs, PUC cell isolates and porcine fibroblasts. Immunocytochemical analysis detected Nanog immunoreactivity in PUC cell nuclei, and faint labeling in fibroblasts. Oct-4 immunoreactivity was detected in the nuclei of some PUC cells, but not in fibroblasts.

**Conclusion:**

Cells isolated from PUC express three transcription factors found in pluripotent stem cell markers both at the mRNA and protein level. The presence of these transcription factors, along with the other characteristics of PUC cells such as their colony-forming ability, Hoechst dye-excluding side population and alkaline phosphatase expression, suggests that PUC cells have properties of primitive pluripotent stem cells. Furthermore, PUC cells are an easily and inexpensively obtained source of stem cells that are not hampered by the ethical or legal issues associated with ESCs. In addition, these cells can be cryogenically stored and expanded.

## Background

Stem cells constitute a population of cells that are present in all stages of development from preimplantation embryos through adulthood. Pluripotent stem cells have the ability to self-renew for indefinite periods in culture and to differentiate, e.g., give rise to all of the specialized cells in an animal. Currently, pluripotent stem cells can be isolated consistently only from the inner cell mass of embryos or from the gonadal ridge [1]. In contrast, multipotent stem cells have the ability to self-renew and to differentiate into two of the three germ layers; this type of cell has been isolated from adult animals [2]. It would be advantageous to have a readily available, low cost source of stem cells in large animal species to facilitate biotechnology, since embryonic stem cells (ESCs) are problematic.

Three transcription factors found in mouse and human ESCs play a central role in the regulation of pluripotency and self-renewal. These factors include the POU (Pit/Oct/Unc) domain-containing protein Oct-4 [3-5], Sox-2 [6] and the homeoprotein Nanog [7,8]. All three transcription factors are expressed in high levels in pluripotent cells and are considered markers of primitive stem cells. The conservation of these three transcription factors across mammalian species is becoming apparent. A recent report suggests that they work in a cooperative fashion to regulate many genes [9].

ESCs, derived from the inner cell mass, and primordial or embryonic germ cells (EGCs), derived from the gonadal ridge, are two examples of pluripotent stem cells. Stem cells with more restricted differentiation potential have been isolated from most postnatal tissues. Postnatal sources of stem cells include bone marrow [10], umbilical cord blood [11-13], vasculature or perivascular space [14-16], placental tissue [17,18] amniotic fluid [19], adipose tissue [20] and skin [21]. In general, Oct-4, Sox-2 and Nanog are not highly expressed in these stem cells and they are not thought to be pluripotent. While stem cells from these sources are not pluripotent or immortal, technical considerations such as ease of collection, collection of a large number of cells, collection for autologous transplantation, and ethical considerations such as collection with informed consent, are important counterpoints. For stem cell applications in biotechnology and agriculture, the ability to collect large number of cells, the collection of cells inexpensively and non-invasively, without risk to the donor, and the ability to cryogenically bank and expand the cells *in vitro *are important considerations. Therapeutically, stem cells from non-embryonic sources have been used clinically and have successfully treated a number of diseases. To advance animal biotechnology, it will be necessary to produce ESCs and EGCs that display germ line transmission in species other than the mouse; so far this has not met with success [22].

The stem cell population in Wharton's jelly of the umbilical cord has properties that make it of interest [23-26]. For example, it is simple to harvest by non-invasive means, provides large numbers of cells without risk to the donor, the stem cell population may be expanded *in vitro*, cryogenically stored, thawed, expanded, genetically manipulated and differentiated *in vitro *[24]. The umbilical cord forms early during gestation, and it encloses the yolk sac, which is the embryologic source of both primordial germ cells and the first hematopoietic stem cells. Importantly, the umbilical cord's physiological functions end at parturition. This structure is embryonic in origin and harvested after birth. The umbilical cord Wharton's Jelly and the umbilical cord blood are non-controversial sources of primitive stem cells that can be collected and stored after birth for therapeutic uses or biotechnology.

Here, we characterize umbilical cord Wharton's Jelly stem cells (called umbilical cord matrix cells) collected from pig. The pig is a useful target for stem cell technology for biomedical models for humans [27] and for agricultural applications. Previously, we showed that after exposure to differentiation signals, cells derived from human or porcine umbilical cord matrix are capable of expressing the morphology and markers of neural cells, and the undifferentiated cells express a variety of characteristics indicative of stem cells, for example, telomerase [24]. Here, we extend that characterization by examining the expression of the transcription factors Nanog, Oct-4 and Sox-2. These factors are of central importance to pluripotency and self-renewal in mouse and human embryonic stem cells [3-8]. In addition, several other characteristics of stem cells are evaluated.

## Materials and methods

### Umbilical cord acquisition and cell procurement

All experimental protocols were approved by the Kansas State University Institutional Care and Use Committee (IACUC #2164). Umbilical cords were collected from newborn (term) or mid-gestation (50–55 days post-conception), male and female pigs obtained from the Kansas State University swine farm. Term umbilical cords were collected immediately after birth and placed in sterile 0.9% saline or Ringer solution (composition in mM: 120 NaCl 25 NaHCO_3_, 3.3 KH_2_PO_4_, 0.83 K_2_HPO_4_, 1.2 CaCl_2 _and 1.2 MgCl_2_) and transported to the laboratory at room temperature. Mid-gestation fetuses and day 14–15 postestrus embryonic discs were collected from sows under general anesthesia using standard veterinary procedures. Once in the laboratory, the cords were rinsed in sterile phosphate buffered saline (PBS, composition in mM: 140 NaCl; 2 KCl; 1.5 KH_2_PO_4_; 15 Na_2_HPO_4_), supplemented with 4 μg/ml amphotericin B, 20 μg/ml gentamicin, 100 U/ml penicillin, and 100 μg/ml streptomycin and placed in a sterile petri dish for further processing. Umbilical cord segments 1–3 cm in length were cut longitudinally to expose the two umbilical arteries and the umbilical vein. The vessels were removed and discarded. The remaining umbilical cord tissue including the Wharton's jelly was diced into 2–5 mm^3 ^explants using single edge razor blades, transferred to 6-well tissue culture plates (Corning Inc., Corning, NY) containing 2 ml of cell culture medium (56% DMEM (low glucose, Gibco-Invitrogen Corporation, Carlsbad, CA), 37% MCDB 201 (Sigma-Aldrich, Inc., St. Louis, MO), 2% fetal bovine serum (FBS, Atlanta Biologicals, Lawrenceville, GA); remaining 5%: 1× insulin-transferrin-selenium supplement (ITS, Sigma-Aldrich, Inc.), 1.5 mg/ml AlbuMax I (Gibco-Invitrogen Corp.), 1 nM dexamethasone (Sigma-Aldrich), 100 μM ascorbic acid 2-phosphate (Sigma-Aldrich, Inc.), 100 U/ml penicillin, 100 μg/ml streptomycin, 0.25 μg/ml amphotericin B (Gibco-Invitrogen), 10 ng/ml recombinant human epidermal growth factor (EGF, R&D Systems, Minneapolis, MN), 10 ng/ml rat platelet-derived growth factor (PDGF, R&D Systems)) and maintained in a 37°C incubator with a 5% CO_2 _atmosphere and saturated humidity. Adherent porcine umbilical cord (PUC) cells were observed 24–48 hrs after plating. Tissue explants were removed from culture and the remaining adhered PUC cells were dissociated with 0.1% trypsin + 1.0 mM EDTA in PBS (trypsin/EDTA, Gibco-Invitrogen) for 3 min. Cells that had detached in this time were subcultured to a new flask at a ratio of 1:5 (denoted passage 1) and the remaining cells were discarded. From this preparation PUC cells were maintained in culture and periodically subcultured, seeded onto coverslips for immunocytochemical analysis, or processed for RNA isolation. For long-term storage, PUC cells were also cryopreserved in a freezing medium consisting of 93% FBS and 7% dimethyl sulfoxide (DMSO, Sigma-Aldrich, Inc.). Early results of the experiments described here from frozen and fresh cells were indistinguishable. Thus, no distinction was made between freshly isolated or frozen PUC cells. Furthermore, no comparisons between PUC cell isolates derived from the two sexes or isolates derived from term or mid-gestation umbilical cord were conducted.

As a control primary cell, fibroblasts were isolated from a 3–4 month old male pig immediately postmortem. The exterior of one ear was thoroughly cleaned with 70% ethanol and ~1 cm^2 ^of skin was excised along with underlying connective tissue using a sterile scalpel blade and placed in sterile PBS. Small pieces of subdermis were placed in 6 well tissue culture plates with 2 ml of growth medium per well and allowed to proliferate. Growth medium consisted of high glucose DMEM (Gibco-Invitrogen) supplemented with 10% FBS, 100 U/ml penicillin and 100 μg/ml streptomycin. Explants were removed within 3 days and the proliferating fibroblasts were allowed to reach confluency. Primary cultures were then processed for immunocytochemistry or RNA isolation as described below.

### Alkaline phosphatase detection

PUC cells from various isolations and passage numbers were grown on glass coverslips or directly on 12 well tissue culture plates for 2–3 weeks. The medium was refreshed every 48 hours. Alkaline phosphatase activity was detected using an Alkaline Phosphatase Substrate Kit (Vector Laboratories, Burlingame, CA) according to the manufacturer's instructions. A blue reaction product following 20 minutes of exposure confirmed alkaline phosphatase activity. Specificity was verified by including 3 μM levamisole (an inhibitor of most forms of alkaline phosphatase) in the substrate solution prior to cell exposure. Cells were then fixed in 4% paraformaldehyde, mounted in DPX (Sigma-Aldrich, Inc.) and photographed using a Nikon CoolPix digital camera (Nikon, Melville, NY) or a QImaging Retiga 1300 digital CCD camera (Roper Industries, Duluth, GA). Images were processed for publication using CorelDRAW (version 10.0) and/or Paint Shop Pro (version 5.01, Corel Corporation, Ottawa, Ontario, Canada).

### Hoechst dye exclusion and flow cytometry

Frozen PUC cells from four different donors (all between passage 5 and 10) were thawed and allowed to grow on 25 cm^2 ^tissue culture flasks until colony formation occurred (1–2 weeks). PUC cells were dissociated in trypsin/EDTA, pelleted by centrifugation, resuspended at approximately 10^6 ^cells/ml in growth medium, and divided into two 1.5 ml microcentrifuge tubes (a small portion of each isolation was reserved for flow sorter calibration). Each tube was incubated with 2.5 μg/ml Hoechst 33342 (Sigma-Aldrich, Inc.) at 37°C with or without 50 μM verapamil (an inhibitor of multi-drug resistance proteins; Sigma-Aldrich, Inc.) for 90 minutes with intermittent mixing. After incubation, cells were pelleted and rinsed twice in ice-cold Hank's Balanced Salt Solution with 2% FBS (HBSS+FBS; Composition in mM: 137 NaCl; 5.4 KCl; 0.4 KH_2_PO_4_; 0.6 Na_2_HPO_4_; 5.5 glucose). Propidium iodide (Sigma-Aldrich, Inc.) was added at 2 μg/ml immediately prior to analysis to detect dead cells. Fluorescence detection was conducted on a FACSVantage SE flow cytometer (BD Biosciences, San Jose, CA) by counting 10,000–40,000 events. The dyes were excited using an ultra violet laser and fluorescence was measured using 485/22 and 660/20 dichroic bandpass filters. Experiments were performed two times with multiple observations in each run. In a separate experiment, cultured porcine fibroblasts were analyzed using the same protocol. Data were analyzed using WinMDI software (version 2.8; Scripps Research Institute, La Jolla, CA). Statistical analysis was conducted to evaluate the effect of verapamil on the percentage of cells with low dye uptake (Hoechst^low ^cells) using a t-test following log transformation of the data. The hypothesis was that verapamil would decrease the % of PUC cells that were Hoechst^low^. A probability for a type I error of 0.05 (p = 0.05) was considered to be statistically significant.

### RNA isolation, RT-PCR and quantitative RT-PCR

Total RNA was collected from PUC isolations after 1–3 weeks in culture, using an RNeasy Mini Kit (Qiagen, Valencia, CA). Briefly, PUC cells were exposed to a lysis buffer supplied with the kit and transferred to the RNeasy column. A DNase step (RNase-free DNase Set, Qiagen) was performed to remove genomic DNA. Following appropriate washings, RNA was eluted from the column in 20–30 μl nuclease-free water. RNA was also isolated from embryonic discs (day 14–15 postestrus, two isolates of 3 pooled discs) and cultured porcine fibroblasts (two isolates) using the same protocol. Expression of various stem cell markers was assessed using a OneStep RT-PCR kit (Qiagen) and gene specific primers. Primers for *Sox-2 *were designed based on highly homologous regions of human, mouse, bovine and/or sheep sequences (due to lack of porcine sequence at the time). Primers were designed using DNASTAR Primer Select (Macintosh version 4.0; DNASTAR, Inc., Madison, WI) or the web-based Primer3 program [28]. These included *Sox-2 *(FWD: 5'-GCCTGGGCGCCGAGTGGA-3'; REV: 5'-GGGCGAGCCGTTCATGTAGGTCTG-3'; annealing temperature = 64°C, expected product length = 443 bp [Genbank: NM_003106]) and Nanog (FWD: 5'-ATCCAGCTTGTCCCCAAAG-3'; REV: 5'-ATTTCATTCGCTGGTTCTGG-3'; annealing temperature = 60°C, expected product length = 438 bp). The *Nanog *primers described in this report were designed based on new porcine sequence information (unpublished data) and encompassed a large segment homologous to the coding region of the human sequence. Primers for *Oct-4 *were synthesized using a published primer sequence [29] that was reported to span an intron (FWD: 5'-AGGTGTTCAGCCAAACGACC-3'; REV: 5'-TGATCGTTTGCCCTTCTGGC-3'; annealing temperature = 60°C). Product size was estimated to be 335 bp, (see [29]). *β-actin *primers were selected as a loading control (FWD: 5'-ATCTTGATCTTCATGGTGCTGGGC-3'; REV: 5'-ACCACTGGCATTGTCATGGACTCT-3'; annealing temperature = 60°C, expected product size = 545 bp) [30]. The reactions were assembled using 200–800 ng of total RNA, 400 μM dNTPs, 400 nM FWD and REV primers, 1 μl enzyme mix and 5 μl buffer in a 25 μl reaction volume. The following PCR protocol was performed: 30 min reverse transcription step at 50–55°C, 15 min denature step at 95°C followed by 35 cycles of 94°C for 30 sec, annealing step for 30 sec and elongation at 72°C for 45 sec with a final extension at 72°C for 10 min using a Techne Touchgene thermocycler (Krackeler Scientific, Albany, NY). Reactions conducted without reverse transcriptase were run in parallel using ExTaq (TaKaRa, Otsu, Shiga, Japan) in place of the OneStep RT-PCR enzyme mix. PCR products were resolved in a 1% agarose gel containing 1 μg/ml ethidium bromide. Images were captured on a FluorChem 8900 imaging system (Alpha Innotech, San Leandro, CA) and processed for publication as described above.

Porcine embryonic disc, PUC cell, and porcine fibroblast RNA was collected as described above and subjected to quantitative RT-PCR using *Nanog*, *Oct-4*, *Sox-2 *and 18S ribosomal subunit primers (18S primer sequences were as follows: FWD: 5'-GAGGTTCGAAGACGATCAGA-3'; REV: 5'-TCGCTCCACCAACTAAGAAC-3'; annealing temperature = 55°C). In two separate experiments, RNA was collected from three pooled porcine embryonic discs (approximately 14 days postestrus), porcine fibroblasts from a single pig, and two different PUC cell isolations. In a third experiment, RNA was isolated from three pooled embryonic discs (approximately 14 days postestrus), porcine fibroblasts from a single pig and one PUC isolation, all of which were collected from different pigs than the first two experiments. RNA was quantified using an ND-1000 spectrophotometer (NanoDrop Technologies, Wilmington, DE) and diluted to nominally 10–200 ng/μl. Quantitative reactions were assembled using a Qiagen OneStep RT-PCR kit, 20, 200 or 800 ng of total RNA and 1× SYBR Green dye (Roche, Indianapolis, IN) in a 25 μl reaction volume. PCR was conducted for 50 cycles with SYBR Green fluorescence detected after each cycle. Reverse transcription was omitted as a negative control by replacing the Qiagen enzyme mix with ExTaq DNA polymerase (TaKaRa). All other parameters were identical to non-quantitative reactions (see protocol above). Reactions were carried out on a SmartCycler system (Cepheid, Sunnyvale, CA). For data analysis, the Ct value (the cycle number at which the fluorescence crosses the threshold) for each of the three markers was normalized to 18S to correct for minor variances in loading. The mRNA copy number relative to fibroblasts was calculated by raising 2 to the power of the difference of embryonic disc or PUC Ct value and fibroblast Ct value, providing a fold-difference to compare the relative expression levels.

Sequencing of PCR products was conducted by excising the bands from the agarose gel and purifying the complementary DNA (cDNA) using a QIAquick PCR purification kit (Qiagen). The purified cDNA was either sequenced at the Kansas State University DNA Sequencing Facility, or further processed for sequencing on a CEQ 8000 DNA sequencer (Beckman Coulter, Fullerton, CA). Briefly, purified cDNA was resolved by gel electrophoresis, prepared for analysis using a dye terminator cycle sequencing kit (DTCS, Beckman Coulter), precipitated and suspended in an appropriate sequencing buffer. The resulting sequences were analyzed and aligned using the Basic Local Alignment Sequence Tool (BLAST) either to known GenBank gene entries or to the porcine database in The Institute for Genomic Research (TIGR). Alignment figures were created using BOXHADE 3.21 .

### Immunocytochemistry

PUC cells were seeded onto glass coverslips and allowed to grow for up to 2 weeks. Porcine fibroblasts were similarly seeded and cultured for up to 7 days prior to analysis. Growth medium was removed and the cells were fixed in 4% paraformaldehyde for 5 min followed by permeation with 0.2% Triton-X-100 (Fisher Chemicals; Fairlawn, NJ) and blocked with an appropriate agent (3% normal goat or donkey serum + 1% bovine serum albumin in PBS). The primary antibodies used were goat anti-Oct-3/4 (Santa Cruz Biotechnology; Santa Cruz, CA; n = 3) diluted 1:50 or 1:100 in 0.5× blocking solution, or rabbit anti-Nanog (Chemicon International; Temecula, CA; n = 5) diluted 1:200 and applied for 1 hr at room temperature or overnight at 4°C. Secondary antibodies (donkey anti-goat IgG-FITC conjugated, diluted 1:200 in PBS (Jackson ImmunoResearch Laboratories, West Grove, PA) or goat anti-rabbit IgG-Alexa-488 conjugated, diluted 1:500 in PBS (Molecular Probes, Eugene, OR)) were applied for 1 hour at room temperature. Cells were further counterstained with 4',6-diamidino-2-phenylindole (DAPI, Vector Laboratories, Burlingame, CA) and imaged on a Nikon Eclipse 80i microscope fitted with a Photometrics CoolSnap ES digital camera using appropriate filters. Images were captured with MetaMorph software (Molecular Devices Corporation, Sunnyvale, CA) and processed for publication using CorelDRAW and/or Paint Shop Pro with identical settings for control and experimental slides.

## Results

### Morphological assessment and selection

**Figure 1:**
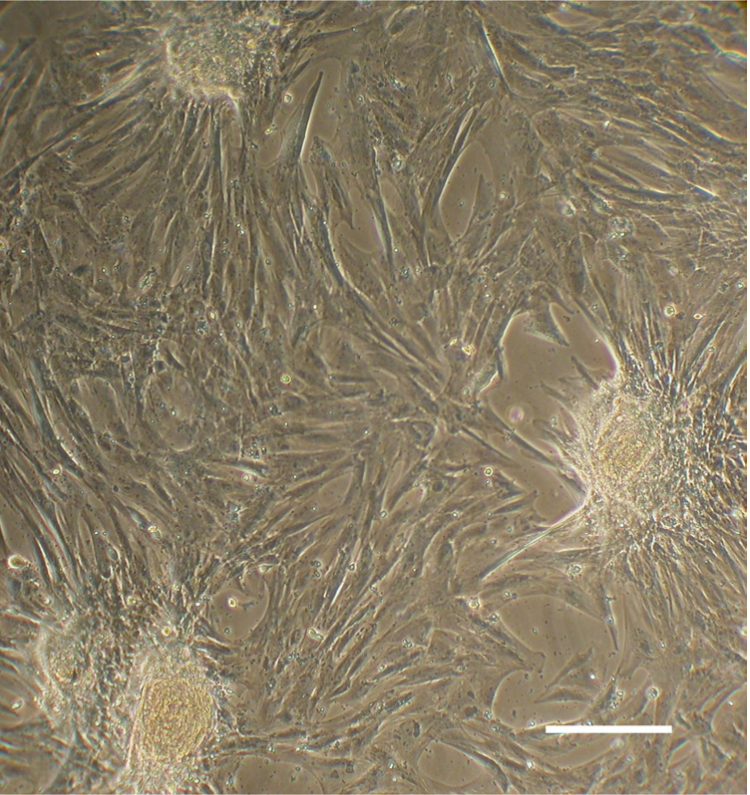
**PUC cells isolated from Wharton's jelly explants form colonies**. Typical morphology of PUC cells after 1–2 weeks in culture. Spindle-shaped PUC cells adhered to plastic growth plates and formed colonies of tightly-packed, small round cells. PUC cells have been maintained in culture for up to 14 passages without noticeable change. Bar = 200 μM.

Cells isolated from porcine umbilical cord matrix explants were expanded in culture and displayed a heterogeneous morphology with many spindle-shaped cells and small round cells with a high nucleus to cytoplasm ratio. The round cells often formed colonies or clusters after 1–2 weeks in culture (Fig. 1). In these studies, approximately 60% (27 of 45) of PUC cell isolations yielded colonies, although all isolates yield cells of similar morphology. Colonies appeared as tightly packed mounds or clusters of small round cells and grew to as much as 0.5 mm in diameter, although most were smaller (0.1–0.2 mm). The formation of colonies was observed in PUC cells as late as passage 14. Data presented in this report were obtained from PUC isolations no later than passage 14.

**Figure 2:**
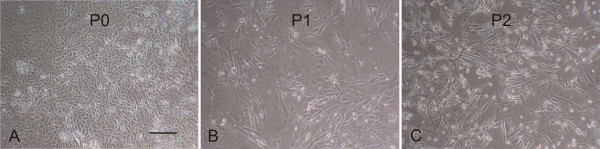
**PUC cells selected for stem cell-like morphology**. The initial population of cells (passage zero, or P0; **A**) expanded from explants contain many different cell types based on morphology. After one passage (P1) using the described selection technique, the number of round and fibroblast-like cells was significantly increased (**B**) and was further enhanced after passage 2 (**C**). Bar = 200 μM.

The isolation of cells with stem cell-like morphology (small, round cells and spindle-shaped cells) was enhanced by selectively eliminating the population of tightly adhered, apparently differentiated cells at the first passage. This was accomplished by first removing any remaining explanted tissue and incubating the remaining cells in trypsin/EDTA for 3 min. Only the cells that detached in this period of time were resuspended in growth medium and seeded to the next flask. This method was repeated for subsequent passages and increased the proportion of cells with stem cell-like morphology as well as their ability to form colonies (Fig. 2). Cells that remained adhered to the flask at passage were large, flat cells that produced few colonies as compared to the passaged cells. These cells were ultimately discarded. All PUC cell isolations providing data for this report were conducted in this manner.

### Alkaline phosphatase detection

**Figure 3:**
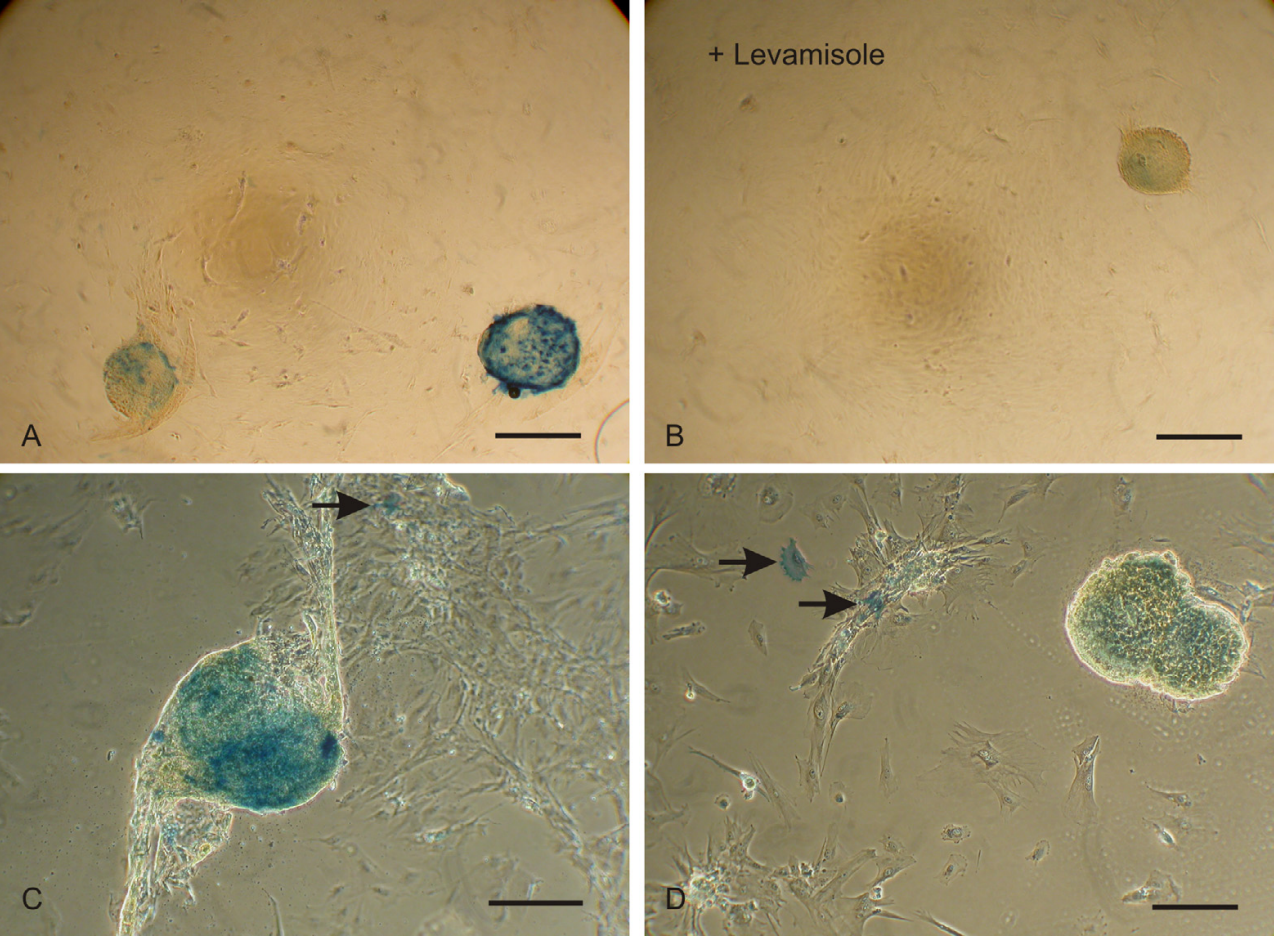
**PUC colonies express alkaline phosphatase**. Colony-forming PUC cell isolates stained for the presence of alkaline phosphatase. Staining was most intense in the colonies (**A**, **C **and **D**). Addition of 3–5 μM levamisole to the solution inhibited the staining (**B**). Usually, a small number of positive individual cells was also stained (arrows in **C **and **D**). Results are typical of 11 separate experiments. Bars = 500 μM (**A **and **B**), 200 μM (**C **and **D**).

The colonies formed by PUC cells in culture exhibited alkaline phosphatase activity, which is consistent with stem cell identity. PUC cells were maintained in culture until colony formation was observed. The cells were then incubated in the detection solution for 20 min and monitored for the development of a blue reaction product indicating alkaline phosphatase activity. The reaction product was most intense within and surrounding the colonies (Fig. 3A, representative from 11 separate observations), although staining was observed in a small number of individual cells (Fig. 3C and 3D). When levamisole was included in the substrate, no reaction was observed (Fig. 3B).

### Hoechst dye-exclusion and flow cytometry

**Figure 4:**
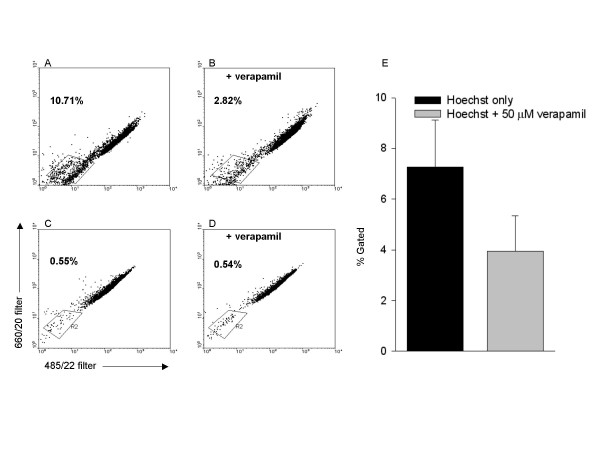
**PUC isolations contain a side population of verapamil-sensitive, Hoechst-dye excluding cells**. A side population of Hoechst^low ^cells in PUC cell isolations (**A**) was visible after using dual-filter flow cytometry, and was partially inhibited with the addition of 50 μM verapamil (**B**). The percent of cells that constituted the side population was in the gated regions. A verapamil-sensitive side population was not observed in porcine fibroblasts (**C **and **D**). A summary of the side population with and without verapamil from four PUC cell isolations is in panel **E**.

As shown in Fig. 4A, flow cytometry revealed a population of Hoechst dye-excluding PUC cells. A distinct population of verapamil-sensitive, Hoechst^low ^cells were identified based on low fluorescence intensity (about 50% of the Hoechst^low ^cells, see Fig. 4B). Five paired experiments (± verapamil) were conducted on four PUC cell isolations. In the absence of verapamil, the percentage of Hoechst^low ^cells was (mean ± SEM) 7.3% ± 1.9% with a range of 3.6–12.7%. Incubation with 50 μM verapamil in conjunction with the Hoechst dye significantly decreased the Hoechst^low ^population to 3.9% ± 1.4% with a range of 1.9% to 9.5% (Fig. 4E). A reduction in the percentage of gated cells was observed in every case in the presence of verapamil. As a comparison, porcine fibroblasts were analyzed in a separate experiment and the percent of Hoechst^low ^cells in the absence and presence of verapamil was not different and the percentage of Hoechst^low ^cells was less when compared to PUC cells (e.g., 0.5% for fibroblasts, see Fig. 4C, D).

### RT-PCR detection and sequence analysis

**Figure 5:**
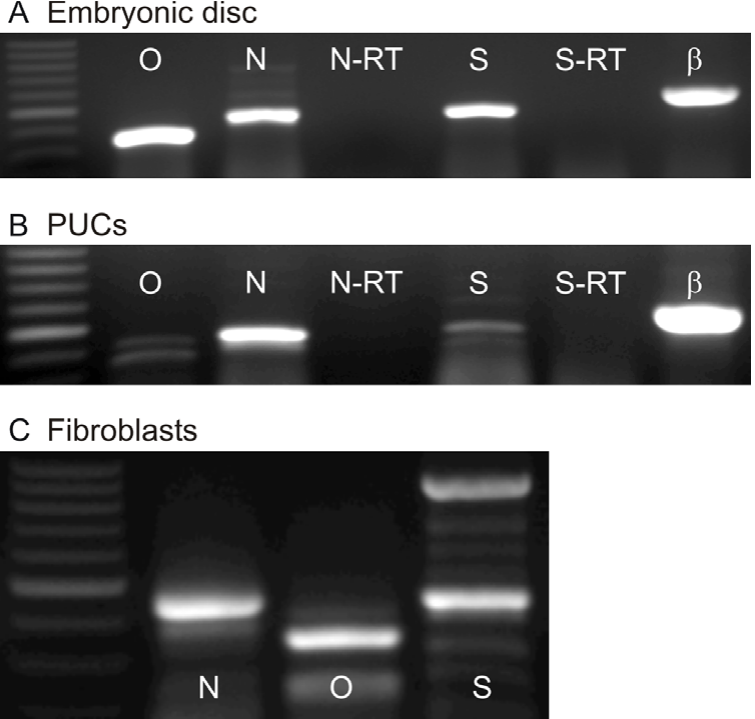
**Transcription factors *Nanog*, *Oct-4 *and *Sox-2 *present in porcine tissue**. Gene specific primers for *Nanog *(N), *Oct-4 *(O) and *Sox-2 *(S) amplified single bands of 438, 335 and 443 bp, respectively, in day 14–15 postestrus embryonic discs (**A**). Bands of expected sizes were also amplified in PUC cells (**B**) as well as in porcine fibroblasts (**C**). Reactions omitting the RT step produced no bands (**-RT**). As a loading positive control, *β-actin *primers (β) were included in most reactions.

Total RNA was isolated from porcine embryonic discs as a positive control for the presence of the three early stem cell markers. Single amplification products of expected sizes were observed using embryonic disc RNA preparations and primers for *Nanog*, *Oct-4 *and *Sox-2 *(Fig. 5A). All three transcription factor genes, *Nanog*, *Oct-4 *and *Sox-2*, were also detected in PUC cells (Fig. 5B). Reactions omitting the reverse transcriptase (-RT) using *Nanog *and *Sox-2 *primers produced no product. The *Oct-4 *primers spanned an intron, therefore the -RT condition was not included. Primers for *β-actin *were included in most experiments as a loading control. Reactions using *Nanog*, *Oct-4 *and *Sox-2 *primers were conducted 41, 46 and 23 times, respectively. These also included primers for *Nanog *and *Oct-4 *that were not described here, but further confirmed the presence of these genes. The lower mobility band for Oct-4 was eliminated by increasing the temperature of the RT step to 55°C (not shown). The band of expected size (Oct-4, 335 bp) was processed for sequencing (see below).

Expression of the three transcription factors was also detected in porcine fibroblasts that were isolated from a 3–4 month old pig (Fig. 5C). The multiple bands observed in the Sox-2 lane in Fig. 5C were observed in 5 of 22 reactions using fibroblasts.

The sequence of *Sox-2 *PCR products employing RNA isolated from PUC cells displayed 96% (388/401 bp) homology to the human sequence [Genbank: NM_003106] and 97% (392/401 bp) homology to the ovine sequence [GenBank: X96997] [see Additional file 2]. Analysis also revealed >99% (400/401 bp) homology to a porcine EST [TIGR: TC208722]. *Nanog *primers produced a single amplified product of expected size from PUC isolations with 86% (326/379 bp) homology to a human *Nanog *sequence [Genbank: AB093576] and a 268 bp segment with 99% homology to the published *Sus scrofa Nanog *partial sequence [GenBank: AY596464] [see Additional file 1]. *Oct-4 *PCR amplified products from PUC isolations displayed >99% (298/300) homology to the *Sus scrofa Oct-4 *sequence [TIGR: TC205936] [see Additional file 3].

### Immunocytochemistry

**Figure 6:**
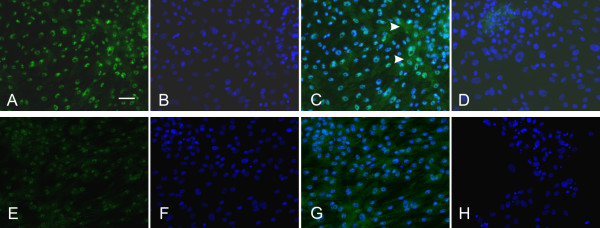
**Nanog protein detection in PUC isolations**. Nanog protein was found in the nuclei of PUC cells (n = 5) (**A**). Comparison to TO-PRO-3 nuclear counterstain (**B**) revealed positive staining in virtually every cell (see superimposed image in **C**), although faint labeling was detected in the cytoplasm of some cells (arrowheads). Superimposing primary antibody-omitted controls with the nuclear counterstain image revealed no protein detection (**D**). Nuclear labeling was detected, but reduced, in porcine fibroblasts (**E**). Superimposition of **E **with nuclear counterstain (**F**) with faint cytoplasmic labeling in some cells (**G**). Primary antibody-omitted control and nuclear counterstain revealed no labeling (**H**). Bar = 50 μM.

Immunocytochemical analysis was performed to test for expression of Nanog and Oct-4 protein. Positive labeling for Nanog in PUC cell nuclei (Fig. 6A) as well as lack of signal when primary antibody was omitted (Fig. 6D), strongly suggests the presence of Nanog protein. Superimposition of images from antibody-treated cells and the DAPI counterstain (Fig. 6B) reveal that virtually every cell displayed nuclear Nanog immunoreactivity (Fig. 6C). Faint cytoplasmic labeling was detected in some cells. Cultured porcine fibroblasts were processed in parallel and revealed very low fluorescence intensity (Fig. 6E). Superimposition with the DAPI counterstain (Fig. 6F) again revealed some cytoplasmic labeling (Fig. 6G). No signal was detected in parallel experiments in which the primary antibody was omitted (Fig. 6H). Experiments were conducted five times.

**Figure 7:**
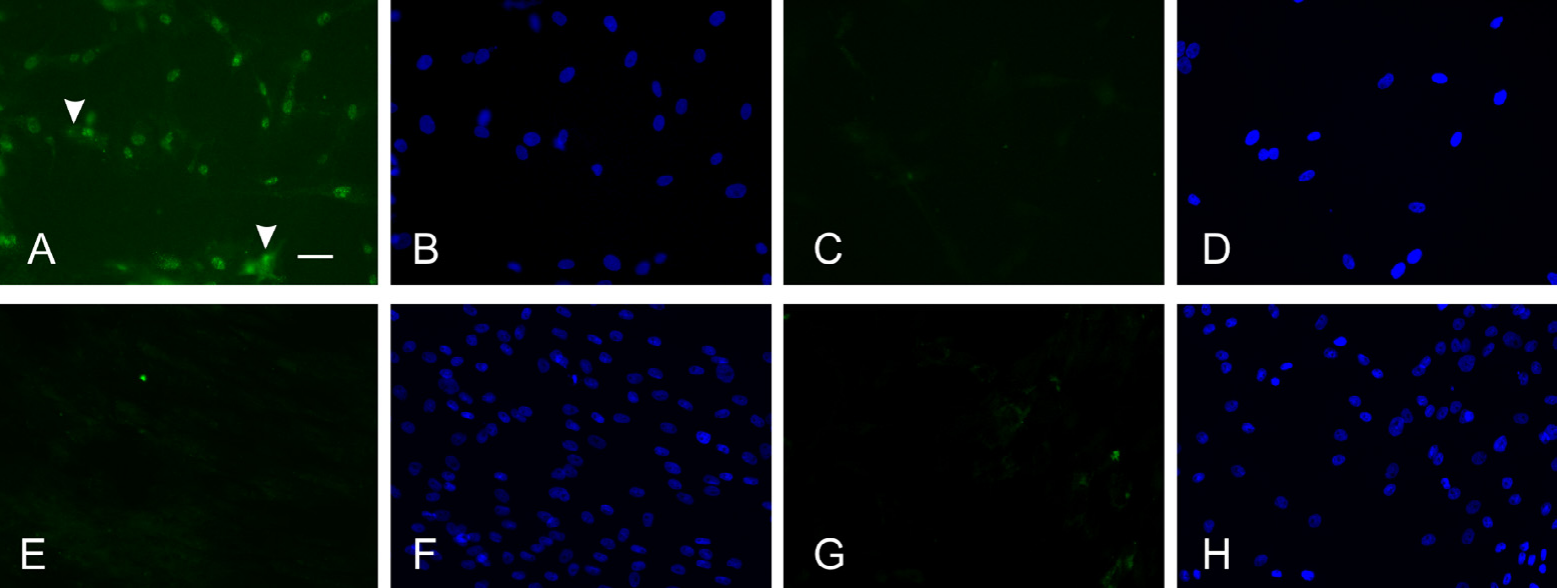
**Oct-4 protein is present in PUC isolations**. Oct-4 detected in the nucleus of PUC cells (n = 3) (**A**). Faint cytoplasmic fluorescence was detected in some cells (arrowheads), this was not found in all PUC isolates. Nuclear counterstaining (**B**) revealed Oct-4 staining in virtually every cell when compared to **A**. No labeling was found when the primary antibody was omitted (**C**). Nuclear counterstain of control shows lack of nuclear Oct-4 staining (**D**). No label was seen in porcine fibroblasts (**E**) or in primary-omitted controls (**G**). The presence of cells was confirmed with nuclear counterstaining (**H**). Bar = 50 μM.

Nuclear immunoreactivity for Oct-4 (Fig. 7A) was also detected in virtually all PUC cells (2 of 3 isolations) when compared with DAPI counterstain (Fig. 7B). Porcine fibroblasts were consistently negative (in two separate experiments, Fig. 7E). DAPI counterstain in Fig. 7F confirms the presence of cells. No fluorescence for Oct-4 was observed in the absence of primary antibody in either cell type (Fig. 7C and 7G), but DAPI counterstain again confirmed the presence of cells (Fig. 7D and 7H).

### Quantitative RT-PCR

**Figure 8:**
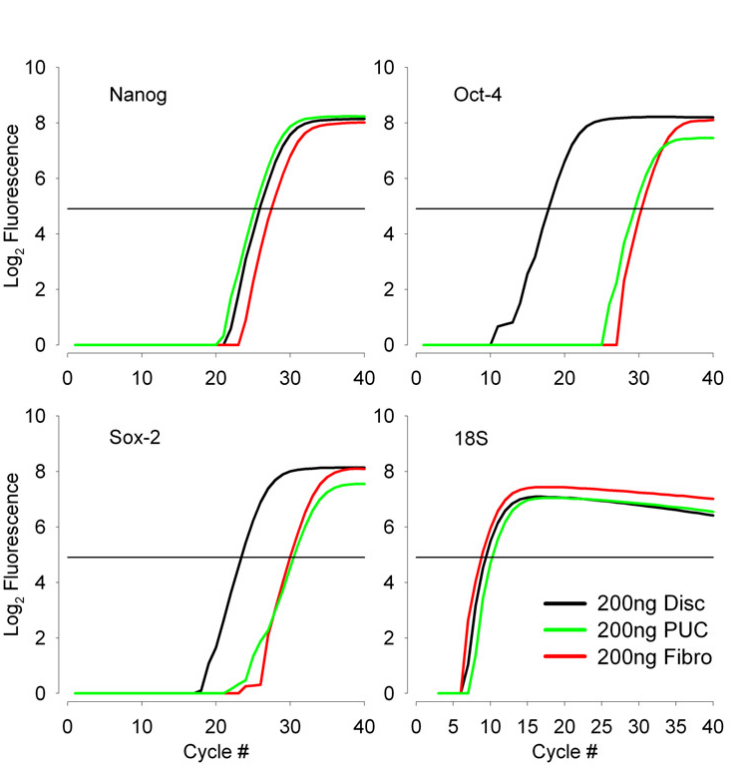
**Quantitative RT-PCR for *Nanog*, *Oct-4 *and *Sox-2 *expression in embryonic disc, PUC cells and fibroblasts**. Known concentrations of total RNA from day 14–15 embryonic discs (Disc, black line), early passage PUC cells (PUC, green line) and porcine fibroblasts (Fibro, red line) were subjected to quantitative RT-PCR using gene specific primers for *Nanog*, *Oct-4*, *Sox-2 *and 18S ribosomal subunit. 200 ng template RNA from each reaction was used here. The cycle number is shown on the X-axis and log_2 _fluorescence is shown on the Y-axis. Horizontal line in each graph indicates the threshold.

Quantitative RT-PCR was conducted to determine the relative expression levels of *Nanog*, *Oct-4 *and *Sox-2 *in embryonic discs, PUC cells and porcine fibroblasts. Results indicate that differences in the expression levels were apparent between cell types (Fig. 8). Results from one experiment using 200 ng of total RNA are presented as representative data from three independent trials. *Nanog*, *Oct-4 *and *Sox-2 *products are shown along with 18S ribosomal subunit RNA (an internal standard for RNA loading). For *Oct-4 *and *Sox-2*, the embryonic disc sample expressed the highest copy number of target RNA (380 to 6400-fold, and 20 to 790-fold greater than fibroblasts, respectively) followed by PUC cells (0.4 to 110-fold, and 1.4 to 3.9-fold), when normalized to the expression levels of fibroblasts. The copy number for *Nanog *RNA ranged from 1.5 to 40-fold and 1.4 to 8.6-fold higher than fibroblasts for embryonic disc and PUC cells, respectively. Importantly, the results show that these stem cell markers are expressed in greater copy number in PUC cells than in porcine fibroblasts, although less than in porcine embryonic disc.

## Discussion

Here we report expression of Nanog, Oct-4 and Sox-2 in cultured PUC matrix cells. These transcription factors are considered key regulators of gene transcription in primitive stem cells. Quantitative RT-PCR revealed that PUC cells and porcine embryonic discs express *Nanog *RNA at higher levels than porcine fibroblasts. Both *Oct-4 *and *Sox-2 *message was present in discs in substantially greater amounts than in PUC cells, and PUC cells contained more message than fibroblasts. Nanog, Oct-4, and Sox-2 have been reported to occupy the promoters of at least 2,260 human genes and these transcription factors co-occupy 353 gene promoters [9]. Thus, these transcription factors regulate pluripotency and self renewal both jointly and individually in human ESCs. Similar data are not available for the pig, but it is likely that these transcription factors regulate central events in stem cell renewal and potency in this species.

We observed gene expression of RNA for all three transcription factors in porcine embryonic discs, PUC cells, and fibroblasts. Therefore, quantitative RT-PCR data on the expression of these transcription factors was undertaken. In particular, the high level of mRNA expression of *Nanog *in both PUC cells and embryonic discs suggests that the regulatory pathways influenced by Nanog in the pig may be similarly activated or inactivated in the embryonic disc and PUC cells. However, the reduced expression of *Oct-4 *and *Sox-2 *in PUC cells relative to the embryonic disc could indicate that genes regulated by these transcription factors may have different expression patterns in these two tissues. Because genetic studies of the functions of these transcription factors are not available in the pig, the meaning of the lower expression of *Oct-4 *and *Sox-2 *mRNA in PUC cells is unknown. The significance of expression, albeit at low levels, of all three transcription factors in fibroblasts is also unclear but may indicate that quantitative regulation is determinative of the differentiation state in this species.

A recent paper described colony formation and Oct-4 expression in fetal murine and porcine fibroblasts, but failed to replicate these findings in adult-derived porcine fibroblasts [29]. Expression of Oct-4 in fetal fibroblasts was only detected when the cells were maintained in 30% calf serum, suggesting that some constituent of the serum must be present in sufficient quantities for expression to be seen. The growth medium used in the present study contains only 2% FBS, although it is supplemented with several other factors, including EGF and dexamethasone. These differences in culture conditions may explain the divergent results. Other differences in early gene expression between the pig and the mouse have also been noted. For example, Oct-4 is expressed in trophectoderm as well as in the inner cell mass of the pig, but only the latter in the mouse [31]. Other reports have suggested that *Nanog *RNA is present in both the embryonic and extraembryonic porcine conceptus tissues at day 15 of pregnancy [32].

The relative levels of mRNA expression for *Nanog*, *Oct-4 *and *Sox-2 *(as compared to fibroblasts) appears to vary in embryonic discs and PUC cells with embryonic discs clearly expressing more *Oct-4 *and *Sox-2*. Numerous factors may explain these results. First, PUC cells and fibroblasts may contain stem cells with similar gene expression levels to those present in embryonic discs, but have a smaller stem cell to non-stem cell ratio than embryonic discs. However, immunocytochemical analysis suggests that while protein levels may differ in PUC cells and fibroblasts, both cell types display nuclear labeling in a uniform manner suggesting that all cells express (or do not express) Nanog and Oct-4 protein at similar levels. Alternatively, posttranslational modifications may account for the reduced expression of Nanog protein and lack of Oct-4 protein in porcine fibroblasts despite the detection of mRNA for both genes. It is unknown whether no protein or an alternative form of the protein is being expressed in fibroblasts. Nonetheless, PUC cells express *Nanog*, *Sox-2 *and *Oct-4 *at the mRNA level with nuclear immunoreactivity for Nanog and Oct-4 being clearly and uniformly present.

The expression of *Nanog*, *Oct-4 *and *Sox-2 *was detected by RT-PCR in PUC cells. The amplification of these transcription factors using RT-PCR techniques produced bands of expected sizes for each PCR product. The subsequent sequencing of the product and high homology to published human and pig sequences is a strong indication of the presence of these genes. Three separate quantitative analysis experiments revealed higher mRNA expression levels of *Nanog *and *Sox-2 *in PUC cells compared to fibroblasts, while two out of three experiments revealed higher levels of *Oct-4 *mRNA in PUC cells compared to fibroblasts. Nuclear immunocytochemical labeling for Oct-4 and Nanog support this result. Recent studies have identified several processed pseudogenes for *Nanog *and *Oct-4 *in the human and mouse genome, including two that have been identified in expressed sequence tags [33,34]. It is currently unknown whether similar pseudogenes exist in pigs and whether they are expressed. However, nuclear immunolabeling suggests the presence of functional Nanog and Oct-4 protein in PUC cells.

Oct-4, Nanog and Sox-2 are central regulators for maintaining a pluripotent state in mouse and human ESCs. In the mouse, artificial *Oct-4 *upregulation results in differentiation of ESCs into extraembryonic mesoderm or endoderm while *Oct-4 *knockout ESCs differentiate into trophectoderm [35,36]. Upregulation of *Nanog *allows mouse ESCs to remain pluripotent without the need for leukemia inhibitory factor (LIF) supplementation in the growth medium [7,8]. Sox-2 is a regulatory transcription factor expressed in early development in many tissues including ESCs [6]. Gene expression was supported by immunocytochemical detection of a Nanog epitope that demonstrated nuclear labeling, consistent with transcription factor localization. Oct-4 immunoreactivity in the nucleus was also detected. In sum, these data suggest that PUC matrix cells constitute a population of stem cells that share some of the phenotypic characteristics of ESCs and also of non-embryonic stem cell populations.

Alkaline phosphatase activity was observed in colony-forming PUC isolations. Alkaline phosphatase is an enzyme long-known to be expressed in ESCs as well as primordial germ cells [37]. The reaction product formed in PUC cell cultures is most intense in and around the colonies, similar to the pattern observed in ESC cultures and other colony-forming stem cells. Alkaline phosphatase is an enzyme expressed in many tissues and alone is not specific for stem cells. However, during development, primordial germ cells (which express alkaline phosphatase) are formed in the yolk sac and migrate through the developing umbilical cord *en route *to their final destination in the gonadal ridge. It is possible that some of these migrating germ cells or their descendents remain in the umbilical cord matrix.

PUC cells displayed a Hoescht^low ^side population that was detected by flow cytometry. Hematopoietic stem cells contain a small population of Hoechst dye-excluding cells (termed side population or SP) when examined by flow cytometry, and this property is inhibited in the presence of verapamil [38,39]. Although verapamil is best known as a calcium channel blocker, it is also used as an inhibitor of P-glycoprotein, a member of the ATP-binding cassette (ABC) transporter superfamily, which is found in hematopoietic stem cells as well as cancer cells [39]. These side population cells exhibit the greatest hematopoietic stem cell activity in repopulation studies [38]. Typically, the percentage of Hoechst^low ^cells in bone marrow samples is less than 1% of the total population. Our observation of approximately 7% (of which, 3% were verapamil sensitive) suggests that a higher concentration of stem-like cells are present in PUC cell isolations.

The present results are consistent with the interpretation that PUC cells represent a stem cell population that expresses the central regulators required for mouse embryonic stem cells. Transplantation studies will be required to define the potential of PUC cells when placed in various *in vivo *environments, but the characteristics identified here suggest that PUC cells will have a wide potential.

We conclude that PUC matrix cells contain unique and primitive cells whose potential is as yet undefined. Ease of collection and propagation, and abundant numbers make PUC matrix cells an attractive candidate as a resource for stem cell biotechnology and biomedical research.

## Supplementary Material

Additional file 1 **Sequence alignment of Nanog PCR products**. Alignment of Nanog PCR product sequence from embryonic disc (Disc), porcine ear fibroblasts (Ear), PUC cells (PUC) and *Sus scrofa Nanog *gene exon 2 and partial coding sequence from GenBank (AY596464). Disc, Ear and PUC sequences share a 268 bp segment with 99% homology to the published *Sus scrofa *sequence. Click here for file

Additional file 2 **Sequence alignment of Sox-2 PCR products**. Direct sequencing of embryonic disc (Day15_Disc) and PUC cells (PUC1) using *Sox-2 *primers were aligned to the human and ovine *Sox-2 *sequence. PUC sequence alignment revealed 96% homology to the human *Sox-2 *sequence and 97% homology to the ovine sequence. Click here for file

Additional file 3 **Sequence alignment of Oct-4 PCR products**. The sequence of two separate PUC isolations using *Oct-4 *primers were aligned to the TIGR *Sus scrofa Oct-4 *sequence (TC205936; TIGR). Alignment revealed 98% (PUC1) and 99% (PUC2) homology. Click here for file
