# Supplementary figures and images for: Expression of early transcription factors Oct-4, Sox-2 and Nanog by porcine umbilical cord (PUC) matrix cells
Source: Reprod Biol Endocrinol. 2006 Feb 6;4:8. doi: 10.1186/1477-7827-4-8 (PMC1373634; doi:10.1186/1477-7827-4-8)

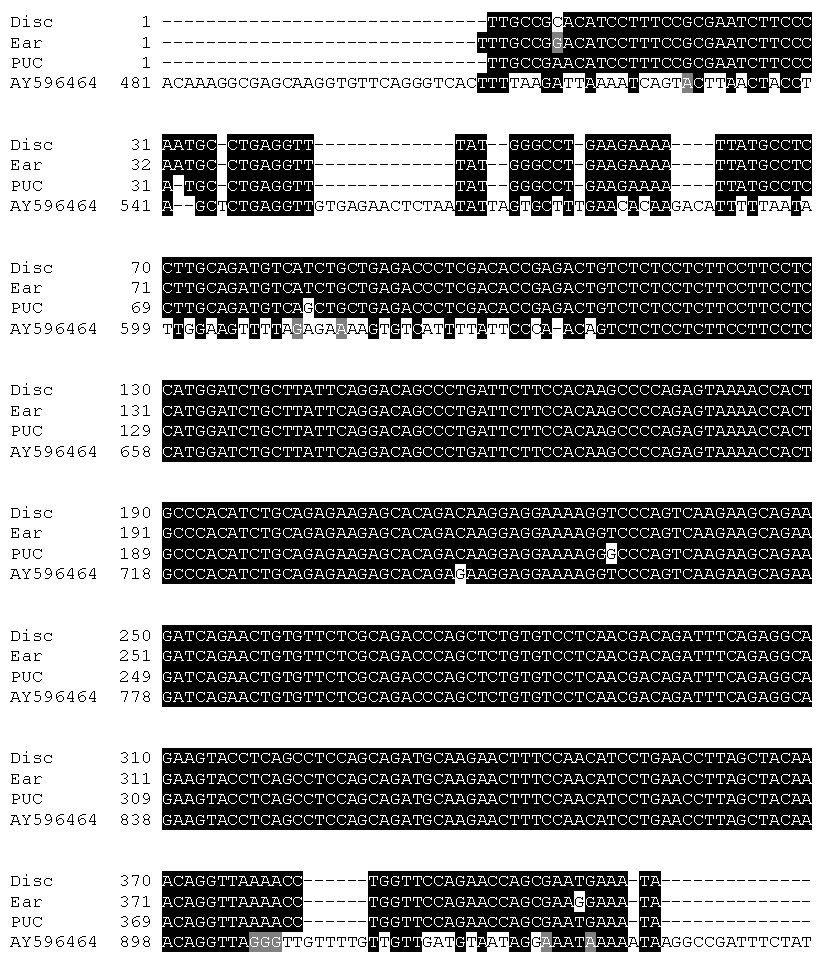

Supplement: Additional file 1 — Sequence alignment of Nanog PCR products. Alignment of Nanog PCR product sequence from embryonic disc (Disc), porcine ear fibroblasts (Ear), PUC cells (PUC) and Sus scrofa Nanog gene exon 2 and partial coding sequence from GenBank (AY596464). Disc, Ear and PUC sequences share a 268 bp segment with 99% homology to the published Sus scrofa sequence. [file 1477-7827-4-8-S1.jpeg]

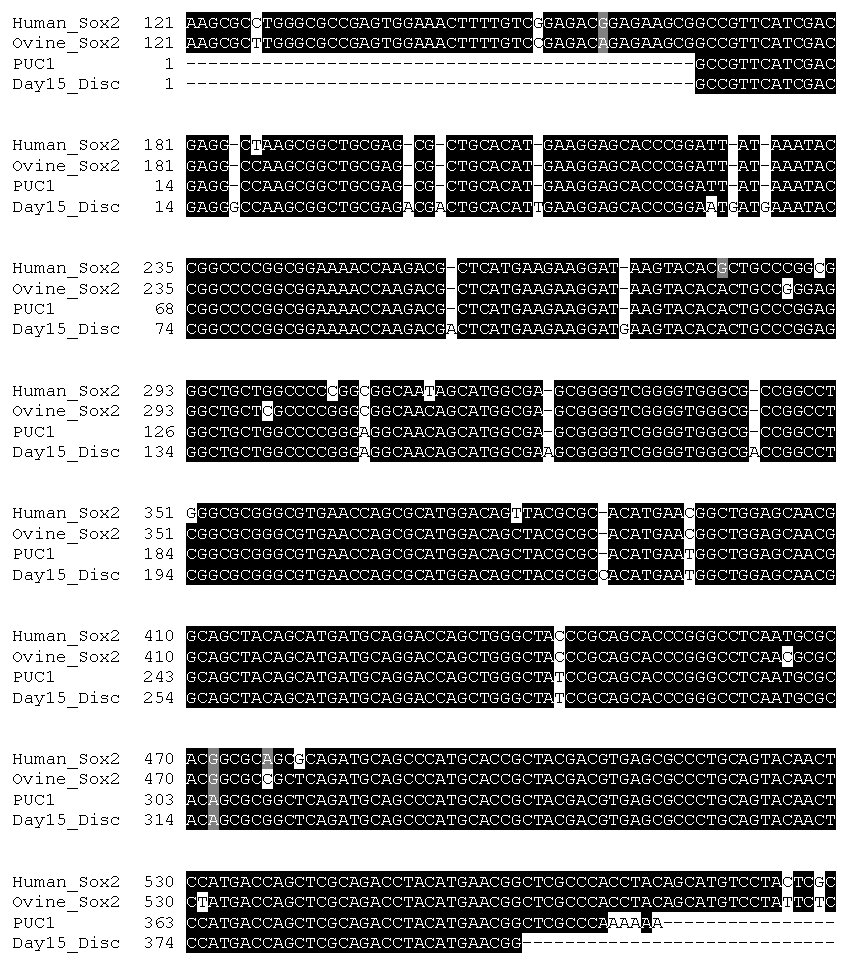

Supplement: Additional file 2 — Sequence alignment of Sox-2 PCR products. Direct sequencing of embryonic disc (Day15_Disc) and PUC cells (PUC1) using Sox-2 primers were aligned to the human and ovine Sox-2 sequence. PUC sequence alignment revealed 96% homology to the human Sox-2 sequence and 97% homology to the ovine sequence. [file 1477-7827-4-8-S2.jpeg]

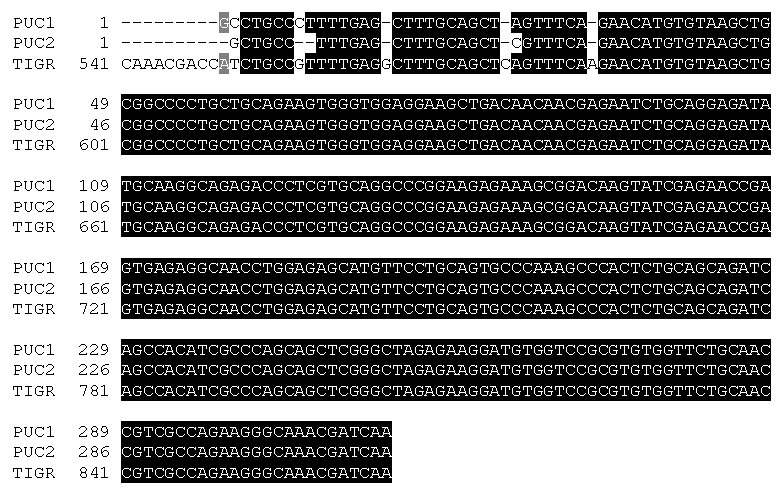

Supplement: Additional file 3 — Sequence alignment of Oct-4 PCR products. The sequence of two separate PUC isolations using Oct-4 primers were aligned to the TIGR Sus scrofa Oct-4 sequence (TC205936; TIGR). Alignment revealed 98% (PUC1) and 99% (PUC2) homology. [file 1477-7827-4-8-S3.jpeg]
